# Hematopoietic stem cell-derived myeloid and plasmacytoid DC-based vaccines are highly potent inducers of tumor-reactive T cell and NK cell responses *ex vivo*

**DOI:** 10.1080/2162402X.2017.1285991

**Published:** 2017-02-06

**Authors:** Soley Thordardottir, Nicolaas Schaap, Elja Louer, Michel G. D. Kester, J. H. Frederik Falkenburg, Joop Jansen, Timothy R. D. Radstake, Willemijn Hobo, Harry Dolstra

**Affiliations:** a Department of Laboratory Medicine - Laboratory of Hematology, Radboud University Medical Center, Nijmegen, the Netherlands; b Department of Hematology, Radboud University Medical Center, Nijmegen, the Netherlands; c Department of Hematology, Leiden University Medical Center, Leiden, the Netherlands; d Department of Rheumatology and Clinical Immunology, University Medical Center Utrecht, Utrecht, the Netherlands; e Laboratory of Translational Immunology, University Medical Center Utrecht, Utrecht, the Netherlands

**Keywords:** Antitumor immunity, dendritic cells, graft-versus-tumor immunity, hematopoietic stem and progenitor cell, mDC, NK cell, pDC, T cell, vaccination

## Abstract

Because of the potent graft-versus-tumor (GVT) effect, allogeneic stem cell transplantation (alloSCT) can be a curative therapy for hematological malignancies. However, relapse remains the most frequent cause of treatment failure, illustrating the necessity for development of adjuvant post-transplant therapies to boost GVT immunity. Dendritic cell (DC) vaccination is a promising strategy in this respect, in particular, where distinct biologic functions of naturally occurring DC subsets, i.e. myeloid DCs (mDCs) and plasmacytoid DCs (pDCs), are harnessed. However, it is challenging to obtain high enough numbers of primary DC subsets from blood for immunotherapy due to their low frequencies. Therefore, we present here an *ex vivo* GMP-compliant cell culture protocol for generating different DC subsets from CD34^+^ hematopoietic stem and progenitor cells (HSPCs) of alloSCT donor origin. High numbers of BDCA1^+^ mDCs and pDCs could be generated, sufficient for multiple vaccination cycles. These HSPC-derived DC subsets were highly potent in inducing antitumor immune responses *in vitro*. Notably, HSPC-derived BDCA1^+^ mDCs were superior in eliciting T cell responses. They efficiently primed naïve T cells and robustly expanded patient-derived minor histocompatibility antigen (MiHA)-specific T cells. Though the HSPC-pDCs also efficiently induced T cell responses, they exhibited superior capacity in activating NK cells. pDC-primed NK cells highly upregulated TRAIL and possessed strong cytolytic capacity against tumor cells. Collectively, these findings indicate that HSPC-derived DC vaccines, comprising both mDCs and pDCs, may possess superior potential to boost antitumor immunity post alloSCT, due to their exceptional T cell and NK cell stimulatory capacity.

**Abbreviations**
AA: Ascorbic acid
AlloSCT: Allogeneic stem cell transplantation
CTL: Cytotoxic T lymphocyte
DC: Dendritic cell
GMP: Good Manufacturing Practice
GVHD: Graft-vs.-host disease
GVT: Graft-vs.-tumor
HS: Human serum
HSPC: Hematopoietic stem and progenitor cell
HSPC-DC: HSPC-derived DC
IFN: Interferon
IL: Interleukin
MiHA: Minor histocompatibility antigen
mDC: Myeloid DC
MoDC: Monocyte-derived DC
PBMC: Peripheral blood mononuclear cell
pDC: Plasmacytoid DC
Poly I:C: Polyinosinic:polycytidylic acid
RPI:C: R848 + Poly I:C
SR1: StemRegenin 1
TLR: Toll-like receptor.

## Introduction

Allogeneic stem cell transplantation (alloSCT) can be a curative treatment of patients with hematological malignancies. The therapeutic effect, the so called graft-versus-tumor (GVT) effect, can be attributed to donor-derived CD8^+^ T cells directed against antigens expressed on the recipient tumor cells, including minor histocompatibility antigens (MiHAs) and tumor-associated antigens (TAAs).^1,2^ In addition, multiple reports demonstrate that NK cells also contribute to GVT immunity, as a high number of NK cells in stem cell grafts,^3^ as well as early NK cell repopulation,^4,5^ have been associated with reduced relapse rates. Still, tumor relapse remains a leading cause of treatment failure, illustrating the necessity to develop potent adjuvant post-transplant immunotherapies to boost GVT immunity, and improve clinical outcome after alloSCT.

Dendritic cell (DC) vaccination is an attractive approach to induce and boost GVT responses, as DCs are the most powerful antigen-presenting cells.^6,7^ Exploiting DC vaccines, T cell responses directed against hematopoietic-restricted MiHAs or TAAs can be specifically boosted, thereby enhancing GVT activity without evoking potentially life-threatening graft-versus-host-disease (GVHD). *In vivo*, multiple blood DC subsets exist, each with their distinct phenotypic and functional characteristics.^8,9^ These subsets can broadly be divided into two groups, myeloid and plasmacytoid DCs (mDCs and pDCs, respectively), where mDCs can be further subdivided into BDCA1^+^ mDCs and BDCA3^+^ mDCs.^10^ mDCs are in the front line of defense against fungi and bacteria, which they detect via different pattern-recognition receptors, including Toll-like receptors (TLRs). They possess high antigen processing and presenting capacity and secrete interleukin (IL)-12, which makes them highly effective in promoting proinflammatory interferon (IFN)-γ secreting T helper (Th)-1 and cytotoxic T lymphocyte (CTL) responses.^8,9,11,12^ In contrast, pDCs play a key role in the detection and control of viral infections, primarily due to their specialized production of type I IFN (IFN-α/β) following TLR7 and TLR9 stimulation.^13^ Their secretion of IFN-α provides an immunostimulatory environment for both innate and adaptive immune cells. Notably, IFN-α and contact-dependent interactions potentiate DC-mediated activation, IFN-γ secretion and cytolytic capacity of NK cells.^14-18^ Furthermore, studies have indicated that the different DC subsets act synergistically via cross-talk, thereby potentiating each others' stimulatory capacity.^19-23^ Together, these characteristics render vaccines composed of both mDCs and pDCs highly attractive for the induction of broad antitumor immune responses.

Recently, we reported an innovative *ex vivo* culture protocol to efficiently generate high numbers of functional BDCA1^+^ mDCs, BDCA3^+^ mDCs and pDCs from CD34^+^ hematopoietic stem and progenitor cells (HSPCs).^24^ We discovered that inhibition of the aryl hydrocarbon receptor, using the antagonist StemRegenin 1 (SR1), was essential for DC subset differentiation. In this paper, we describe optimizations of this SR1-based culture protocol, where culture conditions were improved for generation of higher amounts and better differentiated mDC and pDC subsets. In addition, culture conditions were adapted to Good Manufacturing Practice (GMP). Notably, this protocol allows the generation of the different DC subsets from CD34^+^ HSPCs derived from the original donor grafts. Importantly, we demonstrated the superior capacity of HSPC-derived mDCs and pDCs in stimulating tumor-reactive T cells and NK cells. While the generated mDCs were better T cell stimulators, the cultured pDCs were superior in inducing cytotoxic NK cell responses. Cumulatively, HSPC-derived DC subset (HSPC-DC) vaccines hold great promise for future application as post-transplant therapy to selectively boost GVT immunity and improve relapse-free survival.

## Results

### A GMP-compliant cell culture protocol for generation of high numbers of BDCA1^+^ mDCs and pDCs from HSPCs

**Figure 1.**
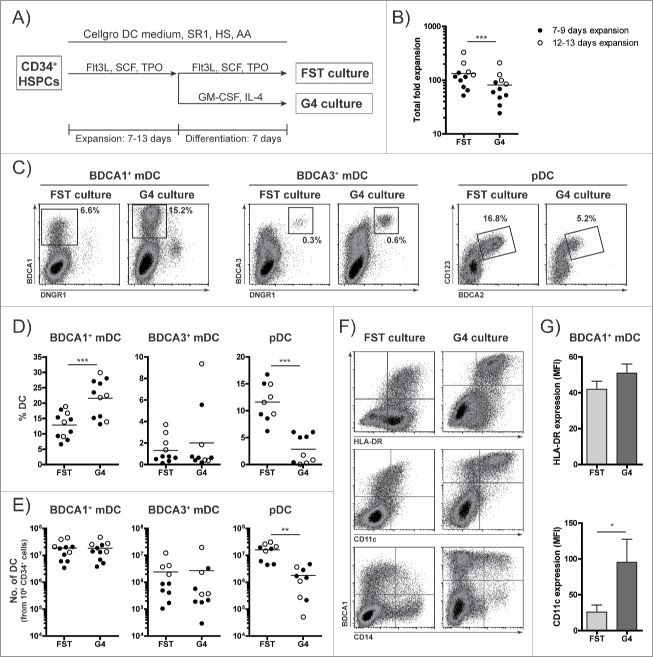
Generation of high numbers of BDCA1^+^ mDCs, BDCA3^+^ mDCs and pDCs from HSPCs. G-CSF mobilized CD34^+^ HSPCs (either freshly isolated or thawed) were cultured for 14–20 d in Cellgro DC medium supplemented with 1 µM SR1, 2% HS and 50 µg/mL AA in a one- or two-step protocol. In the one-step protocol, HSPCs were cultured for 14–20 d in the presence of Flt3L, SCF and TPO (FST), while in the two-step protocol, HSPCs were expanded for 7–13 d with FST, then washed and reseeded in medium containing GM-CSF and IL-4 (G4) and subsequently cultured for seven additional days. (A) Schematic overview of the two different culture protocols, the FST culture (one-step protocol) and G4 culture (two-step protocol). (B) Cumulative fold expansion of total cells at the end of 14–20 d culture, where cells were expanded for 7–9 d (filled circles) or 12–13 d (open circles), followed by 7 d differentiation. Each dot represents an independent donor, lines indicate the mean value (n = 11). (C–E) After 14–20 d culture, the frequencies of BDCA1^+^ mDCs, BDCA3^+^ mDCs and pDCs were determined by flow cytometry. (C) Representative plots showing percentage of BDCA1^+^ mDCs, BDCA3^+^ mDCs and pDCs within total cultured cells. Gating strategy is depicted in Fig. S2. (D) The frequencies of BDCA1^+^ mDCs, BDCA3^+^ mDCs and pDCs within total cultured cells of 9–11 different donors. (E) Numbers of BDCA1^+^ mDCs, BDCA3^+^ mDCs and pDCs generated from 1 × 10^6^ CD34^+^ cells. (D–E) Each dot represents an independent donor, lines indicate the mean value (n = 9–11). (F–G) Surface expression of BDCA1, HLA-DR, CD11c and CD14 on HSPC-DCs in FST and G4 cultures was determined by flow cytometry. (F) Dot plots from one representative donor. (G) Mean fluorescence intensity (MFI) of HLA-DR and CD11c within the BDCA1^+^ mDC population. Data are depicted as mean ± SEM of seven independent donors. Statistical analysis was performed using paired student's *t*-test. *p < 0.05, **p < 0.01, ***p < 0.001.

The overall aim of this study was to generate a DC vaccine composed of both mDC and pDC subsets and to examine its potential to boost both antitumor T cell and NK cell responses. The first objective was to establish culture conditions compliant with GMP, where sufficient numbers of well differentiated and functional DC subsets were generated from HSPCs for eventual clinical application. Therefore, we first modified our recently established SR1-based culture protocol, where the different HSPC-DC subsets were generated under serum-free conditions in GBGM medium supplemented with FLT3L, SCF, TPO, IL-6 and SR1.^24^ Our first optimization steps were to omit IL-6 from the cytokine cocktail and to switch to the widely available GMP-compliant Cellgro DC medium from Cellgenix, supplemented with 2% human serum (HS) and ascorbic acid (AA, 50 µg/mL). We observed that IL-6 inhibited *in vitro* DC differentiation, while AA and HS had a positive effect on DC-generation (Fig. S1). As mDCs generated with the previously published protocol showed low CD11c expression and limited IL-12 secretion upon TLR stimulation,^24^ we next investigated whether the HSPC-DC generation protocol would benefit from an additional mDC-differentiation boost at the end of the culture. For this purpose, we compared a two-step protocol, where HSPCs were first expanded with Flt3L, SCF and TPO (FST) for 7–13 d and then differentiated for one week in the presence of GM-CSF and IL-4 (G4 culture), to the standard one-step protocol where HSPCs were cultured for 14–20 d in the presence of FST (FST culture, Fig. 1A). After 14–20 days, FST-cultured cells had expanded 133-fold in average, while G4-cultured cells expanded only 82-fold in average (Fig. 1B). The frequencies of the different DC subsets were determined in both cultures as depicted in Fig. S2. We observed a significant increase in the frequency of BDCA1^+^ mDCs in G4 cultures compared with FST cultures, while pDC differentiation and/or survival was significantly reduced in G4 cultures (Fig. 1C–D). Nevertheless, low frequencies of pDCs were still detectable in short-term expanded G4 cultures, although these pDCs exhibited lower BDCA2 expression than their FST-cultured counterparts (Fig. 1C, Fig. S2D). The absolute number of generated BDCA1^+^ mDCs was similar for FST and G4 cultures, where both protocols resulted in generation of 18 × 10^6^ BDCA1^+^ mDCs in average from only 1 × 10^6^ CD34^+^ HSPCs (Fig. 1E). Notably, the one-step protocol resulted in almost 10-fold higher absolute numbers of pDCs, where in average 16 × 10^6^ pDCs were generated from only 1 × 10^6^ CD34^+^ HSPCs (Fig. 1E). Although of low occurrence, BDCA3^+^DNGR1^+^ mDCs were detected in both cultures (Fig. 1C–E). Important for clinical implementation, we observed that cryopreserved/thawed HSPCs performed equally well in terms of expansion and DC differentiation as freshly isolated CD34^+^ HSPCs (Fig. S3). Flow cytometric analysis showed that BDCA1^+^ mDCs expressed high HLA-DR in both cultures, while the expression of CD11c increased upon G4 culture, suggestive of a more differentiated phenotype (Fig. 1F–G). Notably, the majority of the BDCA1^+^ mDCs were negative for the monocytic marker CD14 (Fig. 1F).

Based on these data, we continued with the G4 culture for the generation of BDCA1^+^ mDCs, and the FST culture for pDC generation. For further characterization, sorted G4-generated BDCA1^+^ mDCs and FST-generated pDCs were matured overnight with TLR-ligands and evaluated for phenotypical maturation and cytokine secretion. Our previous studies on SR1-derived HSPC-DCs showed that the HSPC-mDCs express TLR3, TLR4, TLR7 and TLR8, while the HSPC-pDCs express TLR7 and TLR9.^24^ To optimally stimulate BDCA1^+^ mDCs, we used a combination of the TLR3 agonist Poly I:C and TLR7/8 agonist R848, as simultaneous stimulation with these two agents has shown a synergistic effect.^25,26^ For stimulation of *ex vivo*-generated pDCs, we used either R848, which induces both IFN-α secretion and upregulation of maturation markers, or the TLR9 agonist CpG-A, known to induce secretion of large amounts of IFN-α.^27,28^ Importantly, we observed high IL-12p70 secretion and tumor necrosis factor (TNF)-α by the matured BDCA1^+^ mDCs (Fig. S4A). Additionally, they upregulated costimulatory and HLA molecules upon TLR-stimulation (Fig. S4B). Similarly, the HSPC-derived pDCs showed a mature phenotype and secreted high amounts of IFN-α and TNF-α (Fig. S4A–B). Moreover, when the DCs were stimulated in the bulk of total cultured cells, high levels of IL-12p70 and IFN-α could be detected, indicating that the total bulk culture could potentially be used as a vaccine (Fig. S4C).

In conclusion, these data demonstrate that using the two-step culture, we can generate a higher frequency of well differentiated, IL-12 producing BDCA1^+^ mDCs, while the FST culture is most optimal for generation of high numbers of IFN-α producing pDCs.

### BDCA1^+^ HSPC-DCs efficiently prime naïve MiHA-specific T cells

**Figure 2.**
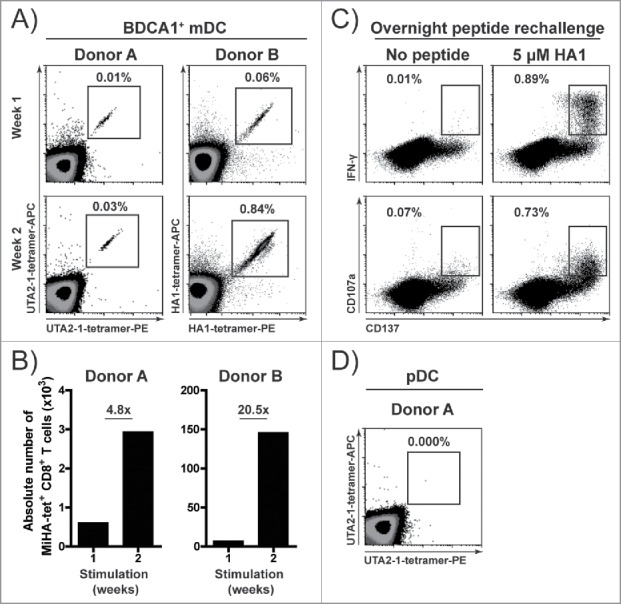
HSPC-derived BDCA1^+^ mDCs efficiently prime and activate naïve MiHA-specific T cells. HSPC-DCs were generated as described in Fig. 1, and subsequently BDCA1^+^ mDCs were sorted from G4 culture and pDCs from FST culture. Next, BDCA1^+^ mDCs were stimulated with R848 and Poly I:C (RPI:C) in the presence of GM-CSF and IL-4, while pDCs were stimulated with R848 in the presence of IL-3. (A–B) Purified CD8^+^ T cells from MiHA-negative HLA-A2^+^ donors were cultured for two consecutive weeks with autologous MiHA peptide-loaded TLR-matured BDCA1^+^ mDCs. Cells were screened for the presence of MiHA-specific CD8^+^ T cells using flow cytometry on days 7 and 14. Density plots (A) and absolute MiHA-specific CD8^+^ T cell numbers (B). (C) BDCA1^+^ mDC-primed and expanded MiHA-specific T cells of donor B were overnight rechallenged with or without 5 µM HA1-peptide at day 14, followed by intracellular staining for CD137, IFN-γ and CD107a. (D) Purified CD8^+^ T cells from MiHA-negative HLA-A2^+^ donor were cultured for one week with MiHA-loaded TLR-matured pDCs. Presence of MiHA-specific CD8^+^ T cells was determined by flow cytometry on day 7. (A, C–D) The numbers in the dot plots represent the percentage of tetramer-positive cells within CD3^+^CD8^+^ T cells.

After establishing the optimal culture conditions for generation of HSPC-DC, we examined their T cell and NK cell stimulatory capacity. To evaluate the capacity of HSPC-DCs in priming naïve antigen-specific T cells, sorted HSPC-derived BDCA1^+^ mDCs and pDCs were matured, loaded with MiHA peptide and co-cultured with autologous purified CD8^+^ T cells from MiHA-negative donors. For these assays, we used three HLA-A2 restricted and hematopoietic-specific MiHAs named HA1^29^, UTA2–1^30^ and HA8.^31^ At day 7, a clear population of MiHA-specific CD8^+^ T cells could be observed upon BDCA1^+^ mDC stimulation (Fig. 2A, Fig. S5), which efficiently expanded upon an additional week of stimulation with MiHA-loaded BDCA1^+^ mDCs (Fig. 2A–B). After overnight peptide rechallenge, BDCA1^+^ mDC-primed MiHA-specific T cells upregulated the activation marker CD137 and were highly positive for both IFN-γ and the degranulation marker CD107a (Fig. 2C). In contrast, no MiHA-specific CD8^+^ T cells could be detected in pDC-stimulated cultures (Fig. 2D). These data demonstrate that the HSPC-derived BDCA1^+^ mDCs are highly effective in priming and activating CD8^+^ T cells from the naïve repertoire.

### BDCA1^+^ HSPC-DCs and pDCs possess superior capacity to expand and activate CD8^+^ T_em_ cells

**Figure 3.**
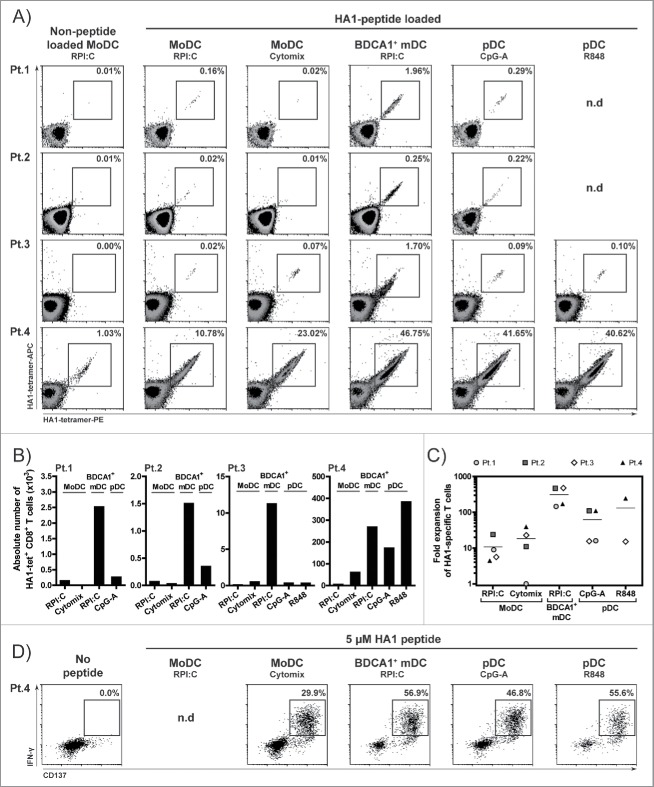
HSPC-derived BDCA1^+^ mDCs show superior capacity to expand and activate antigen-experienced CD8^+^ T_em_ cells. HSPC-DCs were generated as described in Fig. 1, and subsequently BDCA1^+^ mDCs were sorted from G4 culture and pDCs from FST culture. Next, the BDCA1^+^ mDCs, pDCs and autologous MoDCs were activated with respective TLR-ligands as indicated in the figure, in the presence of GM-CSF and IL-4 (MoDCs and BDCA1^+^ mDCs) or IL-3 (pDCs). Thereafter, TLR-matured HA-1 peptide-loaded DCs were co-cultured for one week with patient PBMCs at a 1:0.1 ratio (PBMC:DC) (n = 4). The HSPC-DCs and MoDCs used for expansion of patient HA1-specific CD8^+^ T cells were from unrelated third party donor apheresis products, as there was no apheresis material available of the corresponding transplant donors. The frequency of HA1-specific CD8^+^ T cells at day 0 was <0.02% in Pt. 1–3 and 0.61% in Pt. 4. (A) Density plots showing the percentage of HA1-specific CD8^+^ T cells at day 7. The numbers in the dot plots depict the percentage of positive cells within CD3^+^CD8^+^ T cells. (B) Absolute HA1-specific CD8^+^ T cell numbers after one week co-culture with DCs. (C) Fold expansion of HA1-specific CD8^+^ T cells, calculated by dividing the number of HA1-specific T cells at day 7 with the number of HA1-specific T cells at day 0. (D) 7-d DC-stimulated PBMCs of Pt. 4 were rechallenged overnight with 5 µM HA1-peptide and subsequently stained intracellular for CD137 and IFN-γ. As control, a small portion of cultured cells from all conditions was pooled and cultured overnight without HA1-peptide (No peptide). Numbers in the dot plots indicate the percentage of IFN-γ^+^ CD8^+^ T cells within CD3^+^CD8^+^ T cells. Pt. = patient. N.d. = not determined.

**Figure 4.**
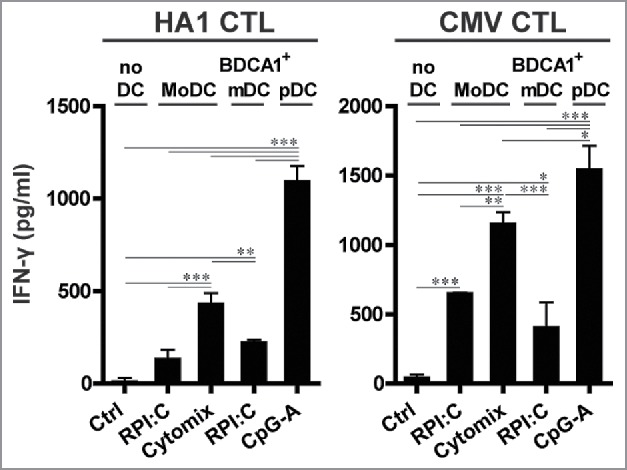
HSPC-derived pDCs show superior capacity to induce IFN-γ secretion by CD8^+^ T_em_ cells. HSPC-DCs were generated and activated as described in Fig. 3. TLR-matured DCs were seeded in triplicate and loaded for 1 h with 1 µM HA1 or CMV peptide at 37°C. Next, without washing, CTLs specific for HA1 or CMV were added at 1:1 ratio and co-cultured for 24 h. Concentrations of IFN-γ were determined by ELISA. Results are depicted as mean ± SD of a representative experiment (HA1 CTL: n = 2, CMV CTL: n = 1). Statistical analysis was performed by one-way ANOVA, followed by Bonferroni post-hoc test *p < 0.05, **p < 0.01, ***p < 0.001.

Next, we assessed the capacity of the HSPC-DC, in comparison to autologous monocyte-derived DCs (MoDCs), to expand and activate effector memory CD8^+^ T (T_em_) cells. In these assays, we used PBMCs (peripheral blood mononuclear cells) from four patients with a hematological malignancy who developed HA1-specific CD8^+^ T cell responses after alloSCT, and stimulated them for one week with TLR-matured HA1 peptide-loaded HSPC-DCs or MoDCs, generated from unrelated third-party HLA-A2^+^HA1^−^ donors. Interestingly, HSPC-derived BDCA1^+^ mDCs and pDCs showed superior capacity to induce expansion of MiHA-specific T_em_ cells, evident by higher frequencies and absolute numbers of HA1-tetramer^+^ CD8^+^ T cells as compared with MoDC stimulation (Fig. 3A–C). In three out of four patients, the BDCA1^+^ mDCs were more efficient than pDCs in boosting T cell expansion. These responses were highly specific, as no expansion of HA1-tetramer^+^ CD8^+^ T cells was seen with non-loaded DCs (Fig. 3A, Fig. S6A). Additionally, the expanded T cells exhibited strong effector functionalities, as reflected by the high percentage of IFN-γ^+^CD137^+^ CD8^+^ T cells from patient four after overnight HA1 peptide rechallenge (Fig. 3D). To explore their T cell stimulatory capacity further, we stimulated established CTL clones with peptide-loaded HSPC-DCs or MoDCs. Interestingly, the pDCs showed better potency for boosting IFN-γ production by HA1 and CMV effector CTLs than mDCs or MoDCs (Fig. 4). Furthermore, TLR-stimulated bulk of total culture HSPC-DCs also efficiently activated HA1 and CMV effector CTLs (Fig. S6B). No IFN-γ secretion was observed in DC:T cell co-cultures in the absence of HA1 or CMV peptide (Fig. S6B). In summary, these data demonstrate the superior capacity of HSPC-derived BDCA1^+^ mDCs in expanding tumor-reactive CD8^+^ T_em_ cells, while the HSPC-derived pDCs appear to exhibit stronger potency to induce effector functions of antigen-experienced T cells.

### HSPC-pDCs superiorly induce activation of cytolytic NK cells

**Figure 5.**
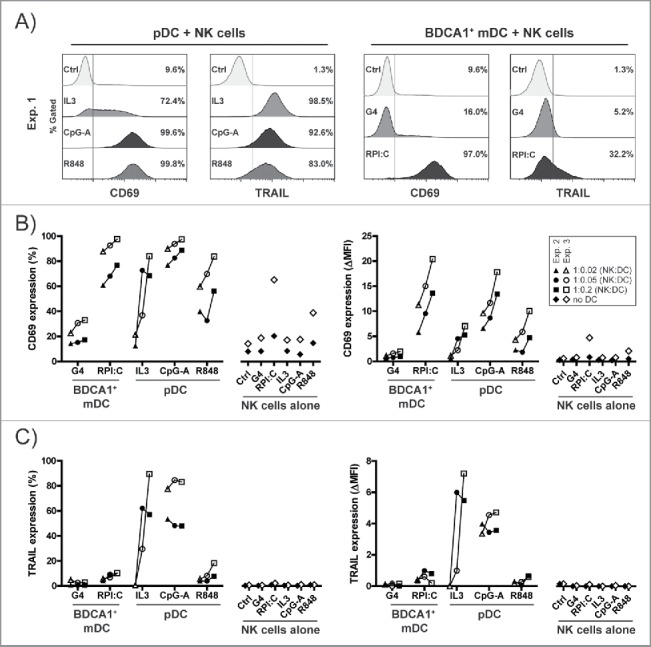
HSPC-pDCs superiorly induce activation of cytolytic NK cells. HSPC-DCs were generated as described in Fig. 1, and subsequently BDCA1^+^ mDCs were sorted from G4 culture and pDCs from FST culture. Next, the different HSPC-DCs were seeded and medium was added containing respective TLR-ligands and cytokines as indicated in the figure. Without washing, allogeneic NK cells were added to the DCs at 1:0.2, 1:0.05 or 1:0.02 ratio (NK:DC). As control, NK cells were cultured without DCs in the absence (control = ctrl) or presence of the different DC maturation cocktails. By flow cytometry, expression of CD69 and TRAIL on NK cells was determined after 40 h co-culture. (A) Histograms depicting expression of CD69 and TRAIL on DC-primed NK cells of experiment 1 at a NK:DC ratio of 1:0.2. (B–C) The percentage and mean fluorescent intensity (MFI) of CD69 (B) and TRAIL (C) on NK cells of experiments 2 and 3 at a NK:DC ratio of 1:0.2, 1:0.05 or 1:0.02. (A–C) Data shown are from three independent experiments using three different HSPC-DC and three different NK cell donors. NK cell purity was 97%, 87% and 97% for experiments 1, 2 and 3, respectively. Exp. = Experiment.

As there is accumulating evidence for the important role of DC-mediated NK cell activation in antitumor immunity,^32-35^ we next assessed the NK cell stimulatory capacity of HSPC-DCs. Therefore, NK cells were co-cultured with sorted allogeneic HSPC-derived BDCA1^+^ mDCs or pDCs in the presence of different DC maturation stimuli. After 40 h of co-culture, a DC dose-dependent increase in CD69 expression on the NK cells was observed, where TLR-matured BDCA1^+^ mDCs and pDCs induced the highest expression (Fig. 5A–B). In contrast, TRAIL expression on NK cells was primarily induced by pDCs (Fig. 5A, C), especially by CpG-A-activated pDCs. TRAIL-expression induced by R848-activated pDCs varied between donors, and was high in experiment 1 (Fig. 5A), but low in experiments 2 and 3 (Fig. 5C). This may possibly have correlated with the respective IFN-α secretion from the stimulated pDCs, which varied in response to R848 (19.5 fg/pDC in experiment 1, <0.1 fg/pDC in experiments 2–3), as studies indicate that TRAIL upregulation on NK cells is dependent upon signaling via type I IFN receptor.^36-38^ Interestingly, NK cells co-cultured at a lower ratio with non-TLR activated pDCs (in the presence of IL-3) also highly expressed TRAIL, despite very limited IFN-α secretion by non-TLR activated pDCs (<0.1 fg/pDC).

**Figure 6.**
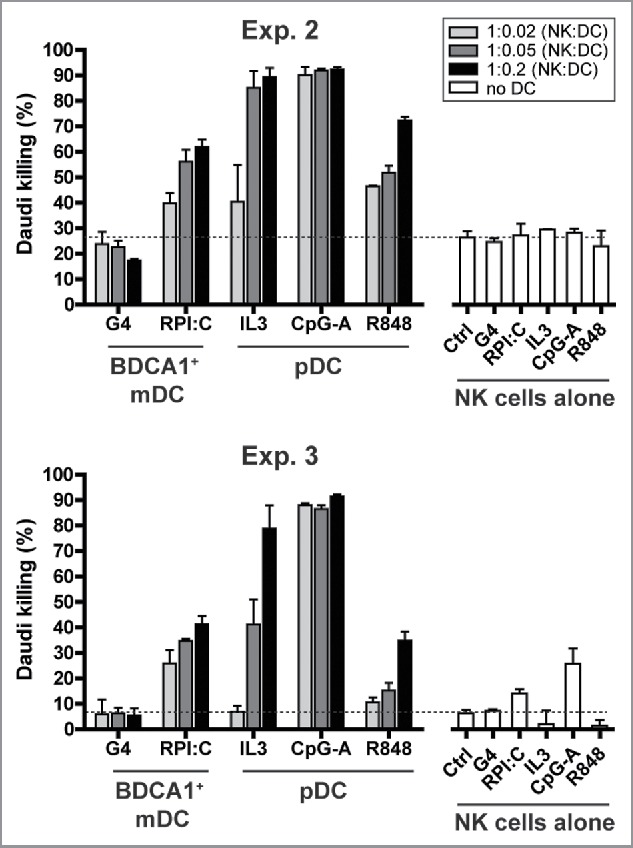
HSPC-pDCs superiorly potentiate cytolytic capacity of NK cells. NK cells were co-cultured with DCs for 40 h as described in Fig. 5. Subsequently, a 4 h flow cytometric cytotoxicity assay was performed, where Daudi tumor cells were added at a NK:Daudi ratio of 1:0.5. The graphs depict percentage of specific killing (mean ± SD of triplicates), and was calculated as follows: 100 – ((absolute number of viable CFSE^+^ Daudi cells cocultured with NK cells/absolute number of viable CFSE^+^ Daudi cells cultured alone) × 100). Data depicted are from the same two experiments as are described in Fig. 5B–C. Exp. = Experiment.

Importantly, NK-mediated cytotoxicity was greatly enhanced following DC priming, in particular with CpG-A-activated pDCs (Fig. 6). NK cells co-cultured with as few as 2% pDCs (ratio 1:0.02) in the presence of CpG-A, killed over 88% of the Daudi cells compared with <30% killing by non-primed NK cells. In addition, correlating with the TRAIL expression, we observed efficient Daudi killing following coculture of NK cells with IL-3-cultured pDCs. In control conditions, where NK cells were cultured without DCs in the presence of the different DC maturation stimuli, expression of CD69 and TRAIL remained low and Daudi killing was also low (Figs. 5B–C, 6). Furthermore, no Daudi killing was observed by DC:Daudi co-cultures in the absence of NK cells (data not shown). These findings show that HSPC-DCs can activate and potentiate cytolytic capacity of NK cells, where in particular pDCs possess superior capability for boosting NK tumor reactivity.

## Discussion

Although alloSCT can be a curative therapy for patients with hematological malignancies, high relapse rates remain the leading cause of treatment failure. DC vaccination using a combination of different DC subsets may be an attractive adjuvant treatment to boost GVT immunity. We and others^39^ postulate that vaccination with multiple DC subsets could not only robustly activate tumor-antigen reactive T cells, but also alloreactive NK cells, the two key effector populations involved in tumor cell elimination post alloSCT. Previously, we reported a novel protocol for the generation of BDCA1^+^ mDCs, BDCA3^+^ mDCs and pDCs from CD34^+^ HSPCs, using the aryl hydrocarbon receptor antagonist SR1 in combination with Flt3L, SCF, TPO and IL-6.^24^ Here, we developed an optimized GMP-compliant culture protocol, where high amounts of mDCs and pDCs can be generated from G-CSF mobilized CD34^+^ cells obtained from alloSCT donors. Notably, omission of IL-6 from the culture, as well as supplementation with AA and HS, had positive impact on *ex vivo* DC differentiation. Furthermore, an additional differentiation boost at the end of the culture, where GM-CSF and IL-4 were added instead of Flt3L, SCF and TPO (named G4 culture) resulted in a higher frequencies of BDCA1^+^ mDCs with high CD11c expression. However, this protocol was less ideal for pDC generation, as GM-CSF and IL-4 inhibited pDC differentiation, as also described by others.^40^ Therefore, we favored generation of HSPC-mDCs using the G4 culture, and generation of HSPC-pDCs using the FST culture. Cumulatively, these changes allow *ex vivo* generation of clinically relevant dosages of HSPC-derived BDCA1^+^ mDCs and pDCs which, importantly, produce high amounts of IL-12 and IFN-α upon TLR stimulation. We envision that these immunostimulatory HSPC-DC, presenting MiHAs and/or TAAs, might be a potent adjuvant therapy for patients with hematological malignancies post alloSCT.

As this HSPC-DC vaccine could be suitable for pre-emptive or therapeutic vaccination post alloSCT, we evaluated their stimulatory capacity. Importantly, we observed DC-subset specific characteristics of our cultured HSPC-DC subsets similar to what has been described in literature.^8-10^ *Ex vivo*-generated BDCA1^+^ mDCs were more efficient in priming naïve T cells and subsequent expansion of MiHA-specific T_em_ cells than pDCs. These characteristics are likely related to the higher co-stimulatory phenotype of BDCA1^+^ mDCs and their secretion of IL-12p70.^8,11^ Previously, we and others have demonstrated that DC-induced MiHA-specific T_em_ cells have efficient killing capacity against malignant cells of hematopoietic origin expressing the respective MiHA.^41-44^ Notably, HSPC-derived pDCs were superior in inducing IFN-γ secretion by T_em_ cells, and additionally promoted superior NK cell activation and cytotoxicity. CpG-A activated pDCs, secreting high levels of IFN-α, showed the strongest NK cell activation. IFN-α is a potent NK cell modulator, and is known to enhance both TRAIL expression and cytolytic capacity of NK cells.^36-38,45^ Interestingly, we observed that non-TLR activated HSPC-pDCs also induced high TRAIL expression and NK cell-mediated cytotoxicity. In the presence of IL-3 alone, no IFN-α was detectable in those co-cultures (data not shown). However, studies indicate that cell-dependent contact with NK cells may enhance the maturation and IFN-α secretion capacity of pDCs.^46^ This may account for the reciprocal activation of NK cells observed.

Nowadays, the majority of alloSCT patients receive hematopoietic stem cells obtained from leukapheresis products of (G-CSF) mobilized donors.^47^ As the yield of CD34^+^ cells is often sufficient for transplantation, it would be possible to cryopreserve ≤ 5% of the donor graft (i.e., max. 3 × 10^9^ total cells or 25 × 10^6^ CD34^+^ HSPCs) for eventual DC vaccine generation. Using the optimized HSPC-DC-generation protocol, the FST culture would result in average in 180 × 10^6^ BDCA1^+^ mDCs and 160 × 10^6^ pDCs from 10 × 10^6^ G-CSF mobilized CD34^+^ HSPCs, and the G4 culture would result in average in 180 × 10^6^ BDCA1^+^ mDCs from 10 × 10^6^ HSPCs. Those achieved numbers are considerably higher than with the previously published protocol,^24^ and could be sufficient for ≥ 9 repetitive vaccinations. Importantly, by generating the DCs from a portion of the donor stem cell graft, these vaccines are obtained without extra apheresis burden for the alloSCT donor. Moreover, during their differentiation, HSPC-DCs can be modified to further augment their stimulatory capacity. For example, immature DCs could be silenced for co-inhibitory molecules such as PD-L1 and PD-L2, by transfection with small interfering RNAs. We have previously demonstrated that PD-L-silenced MoDCs show superior potential in activating tumor-reactive T cells.^48-51^ Additionally, HSPC-DCs could be transfected with mRNA of the tumor target antigen (MiHA or TAA), to provide long-lasting presentation of multiple antigenic epitopes.^49,52-54^

Further pre-clinical studies are warranted to investigate whether enrichment of the different DC subsets will be needed or whether the whole bulk of cultured cells can be used as vaccine. Apart from the different DC subsets generated from the CD34^+^ HSPCs, the culture contained a heterogenous mixture of monocytic and myelocytic cells (defined by CD14, CD15, CD11b and CD34 expression; Fig. S7), but not allogeneic T cells nor B cells (data not shown). Of the total cultured cells, ∼25% and ∼50% within the FST culture and G4 culture, respectively, were HLA-DR negative cells (Fig. S7A–B), which may potentially suppress DC-mediated activation of T cells and NK cells. However, Haar *et al.* recently demonstrated that HSPC-derived HLA-DR^−^ cells obtained with their culture system did not suppress T cell proliferation, but rather induced T cell proliferation.^55^ Within the HLA-DR negative fraction, we could identify a population of cells double positive for CD15 and CD11b (Fig. S7D–E), a phenotype associated with myeloid-derived suppressor cells.^56^ Since we observed these cells primarily in the long (i.e., >19 d) G4 cultures (7–18% of total cultured cells), we favor the shorter culture procedure (i.e., <16 d), where the frequency of HLA-DR^−^CD15^+^CD11b^+^ cells was <4% out of total cultured cells. Shorter culture procedure is also more cost-effective and requires less handling.

It also remains to be investigated which vaccination and TLR stimulation scheme is most optimal for HSPC-DC vaccination. For repeated vaccinations, the HSPC-DCs will need to be cryopreserved. Whether it would be better to cryopreserve, the DCs before or after TLR maturation needs to be established. At the same time, the optimal protocol for TLR stimulation, i.e., which GMP-grade TLR ligands and duration of stimulation, will need to be investigated. Furthermore, there is accumulating evidence that the different DC subsets potentiate each other's stimulatory capacity via cross-talk.^19-23^ For instance, Nierkens *et al.* described that vaccination of mice with mDCs activated in the presence of pDCs resulted in enhanced cross-priming capacity of the mDCs and subsequently augmented antitumor immune responses.^19^ Furthermore, Lou *et al.* demonstrated that immunization of mice with a combination of activated mDCs and pDCs resulted in increased levels of antigen-specific CD8^+^ T cells and facilitated better tumor clearance compared with immunization with either DC subset alone.^20^ It could therefore be favorable to stimulate and/or infuse the different DC subsets simultaneously. However, it could also be more beneficial to harness the distinct features of the different HSPC-DC subsets by independent vaccination with mDCs and pDCs, by infusing the different DC subsets at different locations, and/or at different time-points.

In conclusion, we have developed a GMP-compliant culture protocol where high numbers of different DC subsets, with unique characteristics and functionalities, can be differentiated from the alloSCT donor stem cell grafts. These HSPC-DC subsets are highly potent stimulators of tumor-reactive T cells and NK cells. Together, these findings indicate that vaccination with HSPC-DCs may be an attractive adjuvant therapy post alloSCT to boost GVT immunity.

## Material and methods

### Patient and donor material

For generation of HSPC-DCs and MoDCs, PBMCs were obtained from leukapheresis material of G-CSF mobilized stem cell donors. CD8^+^ T cells used for priming assays were isolated from the same material. For T cell expansion assays, PBMCs were obtained from patients with hematological malignancies who developed HA1-specific CD8^+^ T cell responses following alloSCT.^57^ Samples were collected 6–31 mo after alloSCT. Patient (Pt) 1 and 2 did not receive additional donor lymphocyte infusion post-transplant, whereas Pt 3 and 4 received a donor lymphocyte infusion 16 and 2 mo before the sample date, respectively. Because no apheresis material of the corresponding transplant donors was available, HSPC-DCs and MoDCs used for expansion of these patient-derived HA1-specific T cells were generated from allogeneic HLA-A2^+^HA1^−^ donors (from a third party apheresis). For isolation of NK cells, healthy donor buffy coats were obtained from Sanquin blood bank. Cellular material was obtained in accordance with the Declaration of Helsinki and institutional guidelines and regulations (CMO 2013/064).

### Ex vivo generation of HSPC-DCs and MoDCs

CD34^+^ cells were isolated from G-CSF mobilized PBMCs using anti-CD34 microbeads (Miltenyi Biotec, catalog# 130–046–702). Purity and viability was evaluated by flow cytometry. CD34^+^ HSPCs were either used directly for HSPC-DC generation, or cryopreserved in 50% HS (PAA Laboratories), 42.5% IMDM (GIBCO Invitrogen, catalog# 21980–032) and 7.5% DMSO (Sigma-Aldrich) and thawed at a later time-point for HSPC-DC generation. To generate HSPC-DCs, CD34^+^ HSPCs were cultured for 14–20 d in Cellgro DC medium (GMP-compliant, Cellgenix, catalog# 20801–0500), supplemented with 1 µM SR1 (Cellagen Technology, catalog# C7710–5) as described previously.^24^ The medium was additionally supplemented with 2% HS and 50 µg/mL AA (Centrafarm) during the entire culture period. During the first 7 d of culture, the medium was supplemented with Flt3L, SCF and TPO, 100 ng/mL each (all from Immunotools, catalog# 11343305, 11343328 and 11344865, respectively). For the remaining culture period, the concentrations of SCF and TPO were reduced to 50 ng/mL. Alternatively, HSPC-DCs were generated in a two-step protocol: after 7–13 d expansion as described above, cells were harvested, washed and reseeded in Cellgro DC medium containing 800 IU/mL GM-CSF and 500 IU/mL IL-4 (both from Immunotools, catalog# 11343125 and 11340048, respectively) and cultured for seven additional days. At day 0, the CD34^+^ HSPCs were seeded at 1 × 10^5^–2 × 10^5^ cells/mL, while after day 7 their density was adjusted to 1 × 10^6^–1.5 × 10^6^ cells/mL every 2–3 d with growth factor-supplemented medium. Total number of viable cells was determined by trypan blue exclusion counting at fixed timepoints during the culture period. At the end of the culture, samples were taken for flow cytometric analysis, and absolute numbers of DCs were calculated by multiplying the frequency of DCs with the absolute number of total cells generated from 1 × 10^6^ CD34^+^ cells. For functional experiments, BDCA1^+^ mDCs and pDCs were sorted from HSPC-DC cultures by labeling the cells with anti-BDCA1 (clone L161) and anti-CD123 (clone 6H6) antibodies (both from Biolegend), respectively, followed by fluorescence-activated cell sorting (FACS) using the FACS Aria (BD Biosciences). The sorted DC subsets were in all cases used directly (fresh) for functional assays, except in assays described in Fig. 2A–B, where cryopreserved/thawed DCs were used for day 7 re-stimulation of primed MiHA-specific T cells.

MoDCs were generated in 7 d from plastic adherent monocytes in Cellgro DC medium supplemented with 2% HS, 800 IU/mL GM-CSF and 500 IU/mL IL-4, as described previously.^58^

### DC maturation and cytokine release

Sorted HSPC-DC subsets were resuspended in IMDM supplemented with 10% fetal calf serum (FCS, Integro) and matured overnight with TLR-ligands. pDCs were stimulated with 3.2 µg/mL CpG-A (ODN 2216) or 5 µg/mL R848 (Resiquimod; both from Enzo Life Sciences, catalog# ALX-746–005 and ALX-420–038, respectively) in medium supplemented with IL-3 (10 ng/mL, Immunotools, catalog# 11340035), while BDCA1^+^ mDCs were stimulated with a combination of 5 µg/mL R848 and 20 µg/mL Poly I:C (Polyinosinic:polycytidylic acid, Sigma-Aldrich, catalog# P0913), referred to as RPI:C, in the presence of GM-CSF (800 IU/mL) and IL-4 (500 IU/mL). MoDCs were matured with RPI:C or conventional cytokine mixture (cytomix) containing 5 ng/mL IL-1β, 15 ng/mL IL-6, 20 ng/mL TNF-α and 1 µg/mL PGE2, as described previously.^51^ Cell culture supernatants were analyzed with ELISA for IFN-α (pan-specific, MabTech, catalog# 3425–1H-20), IL-12p70 (eBioscience, catalog# 14–8129 (standard), 16–8126 (coating Ab) and 13–7129 (detection Ab)) and TNF-α (Sanquin, catalog# M9323) levels, according to manufacturer's instructions.

### Flow cytometry

Cells were pre-incubated on ice with total human IgG (Nanogam, Sanquin) to block Fc-receptors, and subsequently stained with appropriate antibody combinations. HSPC-DCs were phenotyped with anti-BDCA1/CD1c (clone L161), anti-BDCA2/CD303 (clone 201A), anti-BDCA3/CD141 (clone M80), anti-BDCA4/CD304 (clone 12C2), anti-DNGR1/Clec9A/CD370 (clone 8F9), anti-HLA-ABC (clone W6/32), anti-CD11c (clone 3.9), anti-CD15 (clone W6D3), anti-CD80 (2D10), anti-CD83 (clone HB15e), anti-CD86 (clone IT2.2), anti-CD123 (clone 6H6, all from Biolegend), anti-HLA-DR (clone Immu357), anti-CD11b (clone Bear1), anti-CD14 (clone RMO52), anti-CD34 (clone 581, all from Beckman Coulter), anti-PD-L1/CD274 (clone MIH1) and anti-CD123 (clone 7G3, both from BD Bioscience). NK cell activation was determined with anti-TRAIL/CD253 (clone RIK-2), anti-CD3 (clone UCHT1), anti-CD56 (clone HCD56) and anti-CD69 (clone FN50, all from Biolegend). Relevant isotype control antibodies were used from Biolegend. MiHA-specific T cells were detected by staining with anti-CD3 (clone UCHT1, Biolegend), anti-CD8^+^ (clone 3B5, Invitrogen) and peptide/MHC-tetramers (HA1.A2: VLHDDLLEA,^29^ UTA2–1.A2: QLLNSVLTL,^30^ HA8.A2: RTLDKVLEV^31^), as described previously.^42,51,59^ Acquisition and data analysis was performed with a Gallios flow cytometer and Kaluza software (both from Beckman Coulter). Dead cells were excluded using Sytox blue (Invitrogen, catalog# S34857), 7AAD (Sigma, catalog# A9400) or eFluor780 fixable viability dye (eBioscience, catalog# 65–0865–14).

### MiHA-specific T cell assays

In T cell priming experiments, CD8^+^ T cells were isolated from MiHA-negative HLA-A2^+^ G-CSF mobilized PBMCs from male donors using CD8^+^ microbeads (Miltenyi Biotec, catalog# 130–045–201). For HA1-specific T cell expansion assays, PBMC samples obtained from alloSCT patients containing HA1-reactive CD8^+^ T cells were used. For these assays, HSPC-DCs and MoDCs were generated from HLA-A2^+^ MiHA^−^ donors. Matured MoDCs, HSPC-derived BDCA1^+^ mDCs and HSPC-derived pDCs were harvested, loaded with 5 µM MiHA-peptide (HA1, UTA2–1 or HA8, from LUMC-IHB peptide facility, the Netherlands or ThinkPeptides), washed and subsequently co-cultured with the isolated CD8^+^ T cells or patient PBMCs in IMDM/10%HS at a ratio of 1:0.2 to 1:0.1 (T cell/PBMC:DC), as described previously.^42,51^ For priming assays, a minimum of 9 × 10^6^ CD8^+^ T cells were used per DC subset. At days 2 and 5, IL-2 (50 U/mL, Proleukin®, Chiron) and IL-15 (5 ng/mL, Immunotools, catalog# 11340155) were added to the co-cultures. At day 7, cells were counted and analyzed by flow cytometry, and when indicated, re-stimulated with the respective DC subset. For re-stimulation, cryopreserved/thawed DCs were used. To determine functionality, DC-stimulated T cells were rechallenged overnight with 5 µM MiHA peptide in the presence of anti-CD107a (BD Bioscience, clone H4A3), followed by an intracellular staining for IFN-γ (BD Bioscience, clone B27) and CD137 (Biolegend, clone 4–1BB), as described previously.^42,51^

### CTL activation

CD8^+^ T cell clones specific for HA1.A2 and CMV.A2 were isolated from patient 4 and a healthy donor, respectively, and expanded following protocols described previously.^60^ Mature DCs were harvested, washed, seeded in triplicate in 96-well round-bottom plate (Corning Costar, catalog# 3799, 10^4^ DCs/well) in IMDM/10%FCS and loaded with 1 µM HA1 or CMV (NLVPMVATV,^61^ LUMC-IHB peptide facility) peptide. After 1 h incubation at 37°C, HA1- or CMV-specific CTLs were added at 1:1 ratio. After 24 h co-incubation, supernatant was harvested and IFN-γ production was measured by ELISA (Thermo Scientific, catalog# M700A (coating Ab) and M701B (detection Ab)).

### NK-DC co-culture

NK cells were isolated using the untouched NK cell isolation kit (Miltenyi Biotec, 130–092–657), resulting in >87% NK cell purity (CD56^+^CD3^−^ cells). HSPC-derived BDCA1^+^ mDCs or pDCs were resuspended in IMDM/10%FCS, whereupon 10 × 10^3^, 2.5 × 10^3^ or 1 × 10^3^ cells were seeded per well in 96-well round-bottom plates. Subsequently, BDCA1^+^ mDCs were matured using GM-CSF/IL-4 or GM-CSF/IL-4/RPI:C, while pDCs were matured with IL-3, IL-3/CpG-A or IL-3/R848, as described above. Next, 5 × 10^4^ freshly isolated allogeneic NK cells were added, resulting in NK:DC ratios of 1:0.2, 1:0.05 or 1:0.02. After 40 h co-culture, CD69 and TRAIL expression on NK cells was evaluated by flow cytometry. Besides, a 4 h cytotoxicity assay was performed with 1 µM CFSE-labeled^62^ Daudi tumor cells in the presence/absence of 40-h DC-primed NK cells at a NK:Daudi ratio of 1:0.2. Subsequently, cells were harvested and stained with 7AAD, followed by quantification of viable Daudi cells using the FC500 flow cytometer (Beckman Coulter). The percentage of specific killing was calculated as follows: 100 – ((absolute number of viable CFSE^+^ Daudi cells cocultured with NK cells/absolute number of viable CFSE^+^ Daudi cells cultured alone) × 100).^62-64^

### Statistics

Statistical analysis was performed using GraphPad Prism 5.0. Student's *t*-test or one-way ANOVA, followed by Bonferroni post-hoc test, was used as indicated. *p*-values <0.05 were considered significant.

## Supplementary Material

KONI_A_1285991_SupplFigs.1-7.docx
