# Supplementary material for: Hematopoietic stem cell-derived myeloid and plasmacytoid DC-based vaccines are highly potent inducers of tumor-reactive T cell and NK cell responses ex vivo
Source: Oncoimmunology. 2017 Feb 6;6(3):e1285991. doi: 10.1080/2162402X.2017.1285991 (PMC5384421; doi:10.1080/2162402X.2017.1285991)
Supplement: KONI_A_1285991_SupplFigs.1-7.docx [file koni-06-03-1285991-s001.docx]

**Supplementary Figure 1**


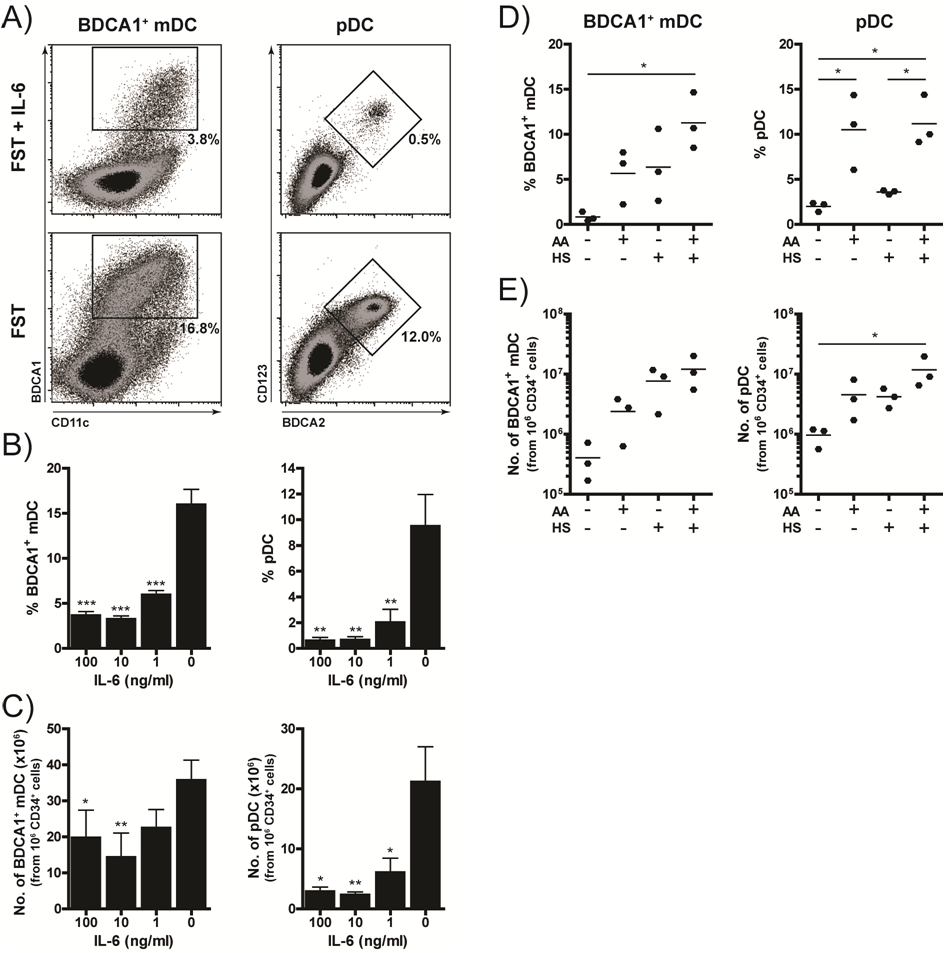


**Supplementary Figure 1. Addition of IL-6 during *ex vivo* culture of CD34^+^ HSPCs inhibits DC differentiation, while supplementation with HS and AA results in improved generation of HSPC-DCs.** A-C) G-CSF mobilized CD34^+^ HSPCs were cultured in Stemspan ACF medium (Stemcell Technologies, catalog# 09855) supplemented with Flt3L, SCF and TPO (FST) and SR1 in the absence or presence of 1, 10 or 100 ng/ml IL-6 (Immunotools, catalog# 11340066). After three weeks culture, the frequency of BDCA1^+^ mDCs and pDCs was determined by flow cytometry. (A) Representative plots showing percentage of BDCA1^+^ mDC and pDC with or without 100 ng/ml IL-6 (n=4). DC are gated from CD14^-^HLA-DR^+^ cells, but numbers in plots show percentage DC within total cultured cells. (B) Frequency within total cultured cells and (C) total yield from 10^6^ CD34^+^ cells of BDCA1^+^ mDC and pDC. (B-C) Data are depicted as mean ± SEM of three independent donors. D-E) G-CSF mobilized CD34^+^ HSPCs were cultured for two weeks in GMP-compliant Cellgro DC medium supplemented with Flt3L, SCF and TPO (FST) and SR1 in the absence or presence of 2% HS and 50 µg/ml AA. Fresh medium containing SR1, FST, HS and AA was added every 2-3 days. At every medium-refreshment, AA was added to a final concentration of 50 µg/ml. After two weeks, the frequency of BDCA1^+^ mDC and pDC was determined by flow cytometry, followed by overnight TLR maturation. D) Frequency within total cultured cells and E) total yield from 10^6^ CD34^+^ cells of BDCA1^+^ mDC and pDC. D-E) Each dot represents an independent donor tested, lines indicate the mean value (n=3). Statistical analysis was performed by using One-way ANOVA, followed by Bonferroni’s multiple comparison test. A-C) Each condition supplemented with IL-6 was compared to FST alone (0 ng/ml IL-6). *p<0.05, ** p<0.01, ***p<0.001

**Supplementary Figure 2**
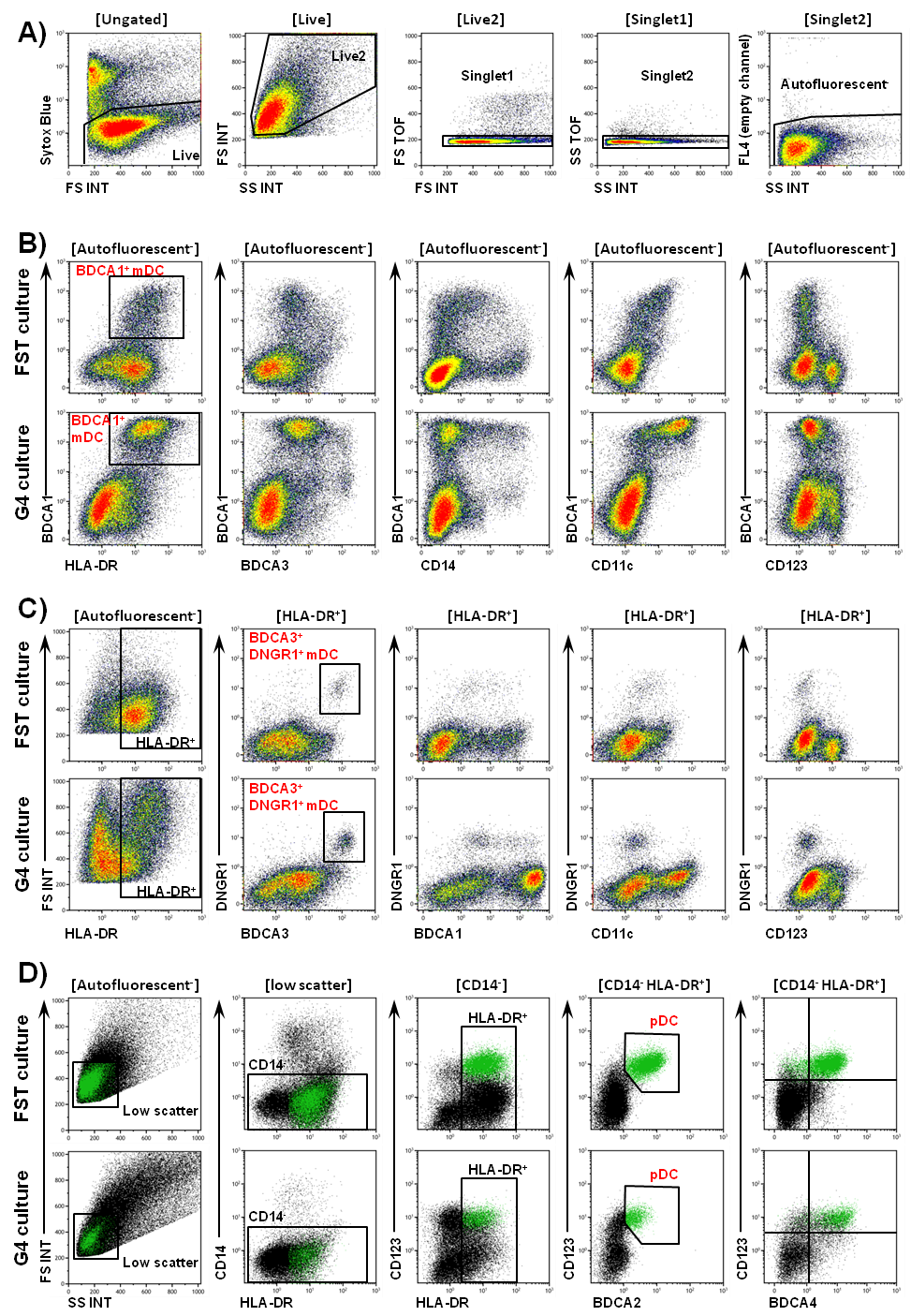


**Supplementary Figure 2. Gating strategy and phenotype of generated HSPC-DCs.** HSPC-DCs were generated as described in Figure 1 and subsequently analyzed by flow cytometry. Density plots from a representative FST culture, where HSPC-DCs were first gated as A) live (Sytox blue^-^, FS versus SS), singlets (FS/SS TOF versus FS/SS INT) and autofluorescent negative (FL4-) cells. The frequencies of the different DC subsets were defined as follows: B) BDCA1^+^ mDCs were defined as BDCA1^+^HLA-DR^+^ cells within autofluorescent negative cells. C) BDCA3^+^ mDCs were defined as HLA-DR^+^BDCA3^+^DNGR1^+^ cells within autofluorescent negative cells. D) pDCs were defined as CD14^-^HLA-DR^+^CD123^+^BDCA2^+^ cells within autofluorescent negative cells with a low FS/SS scatter. Supplementary Figure 2 further depicts the expression of other DC-related markers on the different DC subsets.

**Supplementary Figure 3**


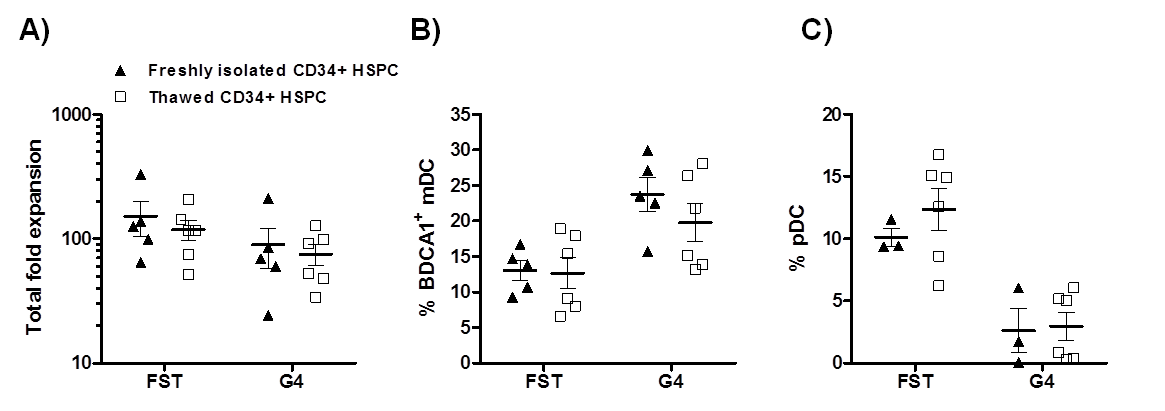


**Supplementary Figure 3. Performance of freshly isolated CD34^+^ HSPCs versus thawed CD34^+^ HSPCs.** HSPC-DCs were generated as described in Figure 1 from either freshly isolated CD34^+^ HSPCs or CD34^+^ HSPCS that were cryopreserved and thawed for later use. Comparison of A) total fold expansion and B-C) frequency of B) BDCA1^+^ mDCs and C) pDCs. Each dot represents an independent donor, lines indicate the mean value ± SEM (n=3-6).

**Supplementary Figure 4**

**
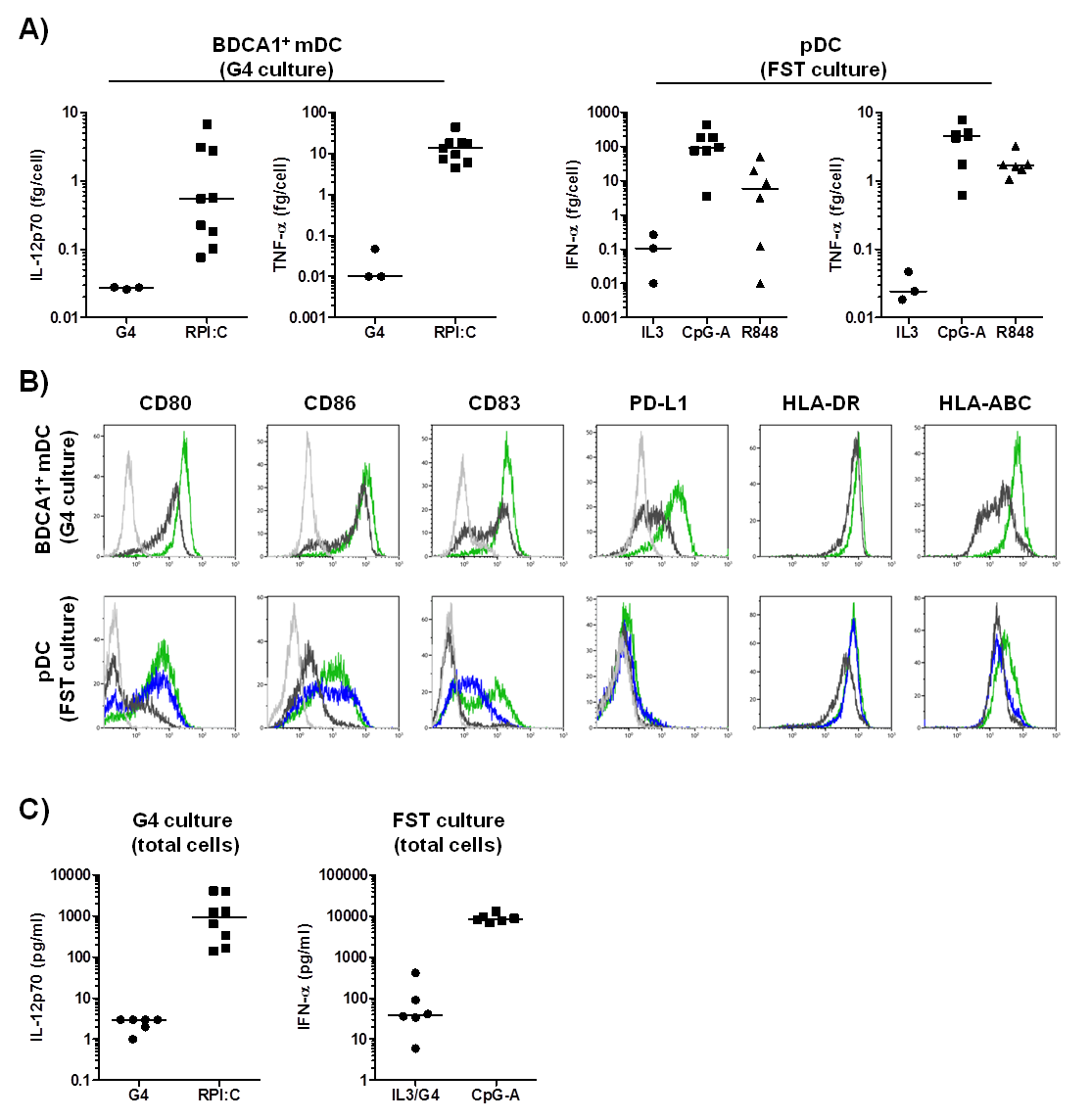
**

**Supplementary Figure 4. Phenotypical maturation and cytokine secretion by sorted CD34-derived DC subsets.** A-B) HSPC-DCs were generated as described in Figure 1, and subsequently BDCA1^+^ mDCs were sorted from G4-culture and pDCs from FST-culture. Next, BDCA1^+^ mDCs were cultured with GM-CSF and IL-4 (G4, black lines)) with or without R848 and Poly I:C (RPI:C, green lines), while pDCs were cultured with IL-3 (black lines) with or without CpG-A (blue lines) or R848 (green lines). After overnight maturation, phenotypical maturation was assessed by flow cytometry and cytokine secretion by ELISA. A) Secretion of IL12p70, TNF-α and IFN-α. Lines indicate median values (n=3-9). B) Histograms of one representative donor show the expression of CD80, CD86, CD83, PD-L1, HLA-DR and HLA-ABC compared to isotype control (grey lines). C) HSPC-DCs were generated as described in Figure 1. Next, total cultured cells were stimulated overnight with TLR ligands at 5x10^5^ cells/ml. Secretion of IL-12p70 and IFN-α was evaluated by ELISA. Lines indicate median values (n=6-8).

**Supplementary Figure 5**


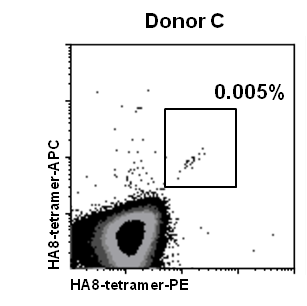


**Supplementary Figure 5. HSPC-derived BDCA1^+^ mDCs efficiently prime naïve MiHA-specific T cells.** HSPC-DCs were generated as described in Figure 1, and subsequently BDCA1^+^ mDCs were sorted from G4 culture. Next, BDCA1^+^ mDCs were stimulated with R848 and Poly I:C (RPI:C) in the presence of GM-CSF and IL-4. Purified CD8^+^ T cells from HA8-negative HLA-A2^+^ donor were cultured for one week with autologous HA8 peptide-loaded TLR-matured BDCA1^+^ mDCs. Cells were screened for the presence of HA8-specific CD8^+^ T cells using flow cytometry on day 7. The number in the dot plot represents the percentage of tetramer-positive cells within CD3^+^CD8^+^ T cells.

**Supplementary Figure 6**

**
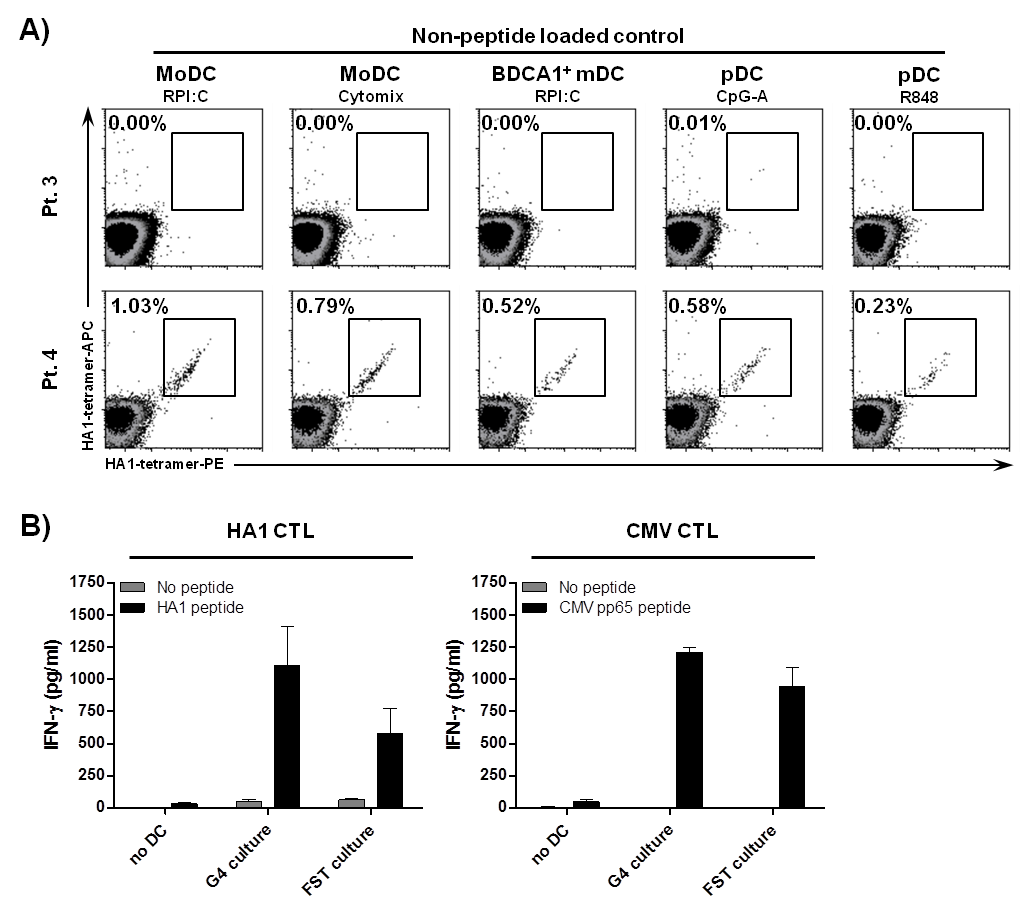
**

**Supplementary Figure 6. CD8^+^ T_em_ cell responses.** A) HSPC-DCs were generated as described in Figure 1, and subsequently BDCA1^+^ mDCs were sorted from G4 culture and pDCs from FST culture. Next, the different DC subsets and autologous MoDCs were activated with respective TLR-ligands and cytokines (GM-CSF and IL-4 (MoDCs and BDCA1^+^ mDCs) or IL-3 (pDCs)) as indicated in the figure. Patient PBMCs containing low frequencies of HA1-specific CD8^+^ T cells were subsequently stimulated with TLR-matured non-peptide loaded DCs at 1:0.1 ratio. After one week stimulation, cells were screened for the presence of HA1-tetramer positive CD8^+^ T cells using flow cytometry. Density plots show tetramer stainings of cultures with PBMCs from Pt. 3 and 4 stimulated with non-peptide loaded DC (the relevant controls for tetramer stains depicted in Figure 3). The frequency of HA1-specific CD8^+^ T cells at day 0 was 0.01% and 0.61% in Pt. 3 and 4, respectively. The numbers in the dot plots represent the percentage of positive cells within the CD3^+^CD8^+^ T cells. Pt. = patient. B) HSPC-DCs were generated as described in Figure 1. Next, total cultured cells were stimulated overnight with TLR ligands and cytokines: G4 culture was stimulated with G4+RPI:C, while FST culture with IL-3+CpG-A. TLR-matured total cultured HSPC-DCs were seeded in triplicate and loaded for 1 hour with 1 µM HA1 or CMV peptide at 37°C. Next, without washing, CTLs specific for HA1 or CMV were added at 1:1 ratio and cocultured for 24 hours. Concentrations of IFN-γ were determined by ELISA. Results are depicted as mean ± SD.

**Supplementary Figure 7**


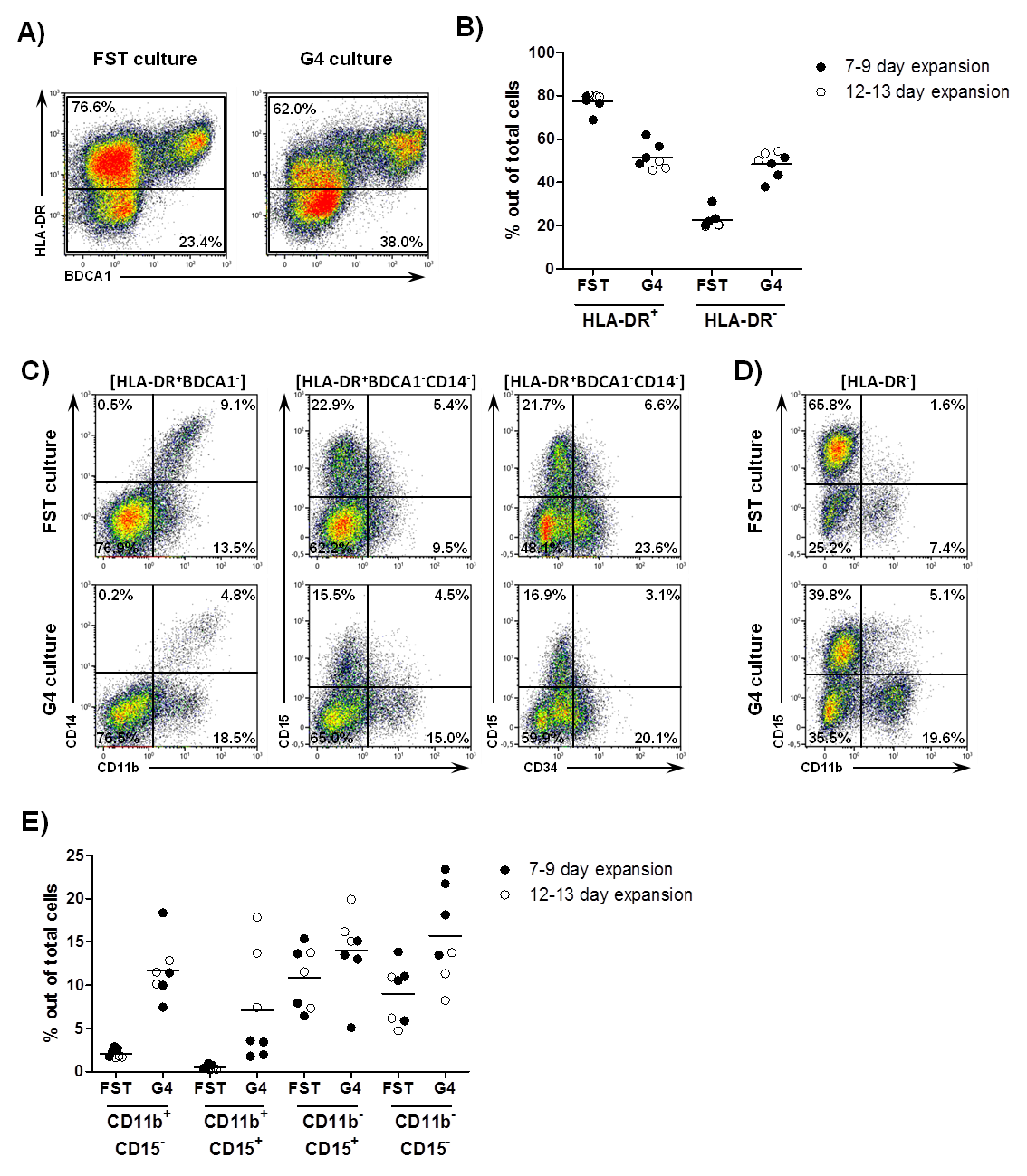


**Supplementary Figure 7. Flow cytometric analysis of HSPC-DC cultures.** HSPC-DCs were generated as described in Figure 1 and subsequently analyzed by flow cytometry for HLA-DR, CD14, CD11b, CD15 and CD34 expression. A) Density plots from one representative donor showing the frequencies of HLA-DR^+^ and HLA-DR^-^ cells within total FST- and G4-cultured cells. B) Frequencies of HLA-DR^+^ and HLA-DR^-^ cells within total FST- and G4-cultured cells of 7 different donors. Lines indicate mean values. C) Density plots from one representative donor depicting expression of CD14, CD11b, CD15 and CD34 within HLA-DR^+^ cells. Numbers in plots indicate percentage positive cells within gated cells (gates are indicated within brackets above FACS plots). D) Density plots from one representative donor showing CD15 and CD11b expression within HLA-DR^-^ cells. Numbers in plots indicate the percentage of positive cells within the HLA-DR^-^ population. E) Frequency of HLA-DR^-^ cells expressing CD15 and CD11b out of total cultured cells. Lines indicate mean values of 7 independent donors.
